# Hypoxia Induces Alterations in the Circadian Rhythm in Patients with Chronic Respiratory Diseases

**DOI:** 10.3390/cells12232724

**Published:** 2023-11-29

**Authors:** Manuel Castillejos-López, Yair Romero, Angelica Varela-Ordoñez, Edgar Flores-Soto, Bianca S. Romero-Martinez, Rafael Velázquez-Cruz, Joel Armando Vázquez-Pérez, Víctor Ruiz, Juan C. Gomez-Verjan, Nadia A. Rivero-Segura, Ángel Camarena, Ana Karen Torres-Soria, Georgina Gonzalez-Avila, Bettina Sommer, Héctor Solís-Chagoyán, Ruth Jaimez, Luz María Torres-Espíndola, Arnoldo Aquino-Gálvez

**Affiliations:** 1 Departamento de Epidemiología e Infectología Hospitalaria, Instituto Nacional de Enfermedades Respiratorias Ismael Cosío Villegas (INER), Mexico City 14080, Mexico; mcastillejos@gmail.com; 2 Facultad de Ciencias, Universidad Nacional Autónoma de México (UNAM), Mexico City 04510, Mexico; yair@ciencias.unam.mx; 3 Red MEDICI, Carrera de Médico Cirujano, Facultad de Estudios Superiores de Iztacala Universidad Nacional Autónoma de México, Mexico City 54090, Mexico; ang-varord@hotmail.com (A.V.-O.); anieetorres@gmail.com (A.K.T.-S.); 4 Departamento de Farmacología, Facultad de Medicina, Universidad Nacional Autónoma de México (UNAM), Mexico City 04510, Mexico; edgarfloressoto@yahoo.com.mx (E.F.-S.); biancasromero_@hotmail.com (B.S.R.-M.); jaimezruth@hotmail.com (R.J.); 5 Laboratorio de Genómica del Metabolismo Óseo, Instituto Nacional de Medicina Genómica (INMEGEN), Mexico City 14610, Mexico; rvelazquez@inmegen.gob.mx; 6 Laboratorio de Biología Molecular de Enfermedades Emergentes y EPOC, Instituto Nacional de Enferdades Respiratorias Ismael Cosío Villegas (INER), Mexico City 14080, Mexico; joevazpe@gmail.com; 7 Laboratorio de Biología Molecular, Departamento de Fibrosis Pulmonar, Instituto Nacional de Enfermedades Respiratorias Ismael Cosío Villegas (INER), Mexico City 14080, Mexico; vicoruz@yahoo.com.mx; 8 Sección de Estudios de Posgrado e Investigación, Escuela Superior de Medicina, Instituto Politécnico Nacional (INP), Mexico City 11340, Mexico; 9 Dirección de Investigación, Instituto Nacional de Geriatría (INGER), Mexico City 10200, Mexico; jverjan@inger.gob.mx (J.C.G.-V.); nrivero@inger.gob.mx (N.A.R.-S.); 10 Laboratorio de Inmunobiología y Genética, Instituto Nacional de Enfermedades Respiratorias Ismael Cosío Villegas (INER), Mexico City 14080, Mexico; ang_edco@yahoo.com.mx; 11 Laboratorio de Oncología Biomédica, Instituto Nacional de Enfermedades Respiratorias Ismael Cosío Villegas (INER), Mexico City 14080, Mexico; ggonzalezavila@yahoo.com; 12 Departamento de Investigación en Hiperreactividad Bronquial, Instituto Nacional de Enfermedades Respiratorias Ismael Cosío Villegas (INER), Mexico City 14080, Mexico; bsommer195@gmail.com; 13 Laboratorio de Neurobiología Cognitiva, Centro de Investigación en Ciencias Cognitivas, Universidad Autónoma del Estado de Morelos, Cuernavaca 62209, Mexico; hecsolch@imp.edu.mx; 14 Laboratorio de Farmacología, Instituto Nacional de Pediatría (INP), Mexico City 04530, Mexico; 15 Departamento de Bioquímica, Facultad de Medicina, Universidad Nacional Autónoma de México (UNAM), Mexico City 04510, Mexico

**Keywords:** circadian cycle, lung diseases, hypoxia, genes, idiopathic pulmonary fibrosis

## Abstract

The function of the circadian cycle is to determine the natural 24 h biological rhythm, which includes physiological, metabolic, and hormonal changes that occur daily in the body. This cycle is controlled by an internal biological clock that is present in the body’s tissues and helps regulate various processes such as sleeping, eating, and others. Interestingly, animal models have provided enough evidence to assume that the alteration in the circadian system leads to the appearance of numerous diseases. Alterations in breathing patterns in lung diseases can modify oxygenation and the circadian cycles; however, the response mechanisms to hypoxia and their relationship with the clock genes are not fully understood. Hypoxia is a condition in which the lack of adequate oxygenation promotes adaptation mechanisms and is related to several genes that regulate the circadian cycles, the latter because hypoxia alters the production of melatonin and brain physiology. Additionally, the lack of oxygen alters the expression of clock genes, leading to an alteration in the regularity and precision of the circadian cycle. In this sense, hypoxia is a hallmark of a wide variety of lung diseases. In the present work, we intended to review the functional repercussions of hypoxia in the presence of asthma, chronic obstructive sleep apnea, lung cancer, idiopathic pulmonary fibrosis, obstructive sleep apnea, influenza, and COVID-19 and its repercussions on the circadian cycles.

## 1. Introduction

The circadian cycle is a biological process present in almost all species, from eubacteria to humans, that governs the synchronicity of both behavior and physiological responses to a light–dark cycle of 24 h [1,2]. This internal rhythm is regulated not only in response to external cues, but also by the internal timekeeping system, a biological clock located in the suprachiasmatic nucleus (SCN) consisting of a group of cells located in the hypothalamus in the brain [1,3]. The SCN receives input from light-sensitive cells in the eyes, which help synchronize the circadian rhythm with the external environment. This biological “clock”, present in both the central regulatory neurons and peripheral tissues, such as the liver, lungs, and skeletal muscle [4], allows tissues and organs to “do the right thing” at the correct time, producing daily oscillations in endocrine processes involved in metabolism, growth, and reproduction, with fluctuating levels of steroidal hormones, peptides, and signaling pathways [1].

This generated information couples the circadian rhythms with physiological adaptations, regulating core temperature, sleep/wake rhythms, immunity, antioxidant activity, hemostasis, and glucose homeostasis [5,6]. The is no specific origin of the study of biological rhythms, i.e., chronobiology, which encompasses the study of biological timing, such as high-frequency, daily, monthly, and annual cycles [1].

It could be considered that the pioneering research that opened the chronobiology field in the 1950s was the circadian rhythmicity study by Colin Pittendrigh in fruit flies and by Jürgen Aschoff in humans [1]. A known marker of the establishment of the circadian rhythm is the initiation of the production of certain regulatory hormones that regulate the rhythm of biological processes, including melatonin, which starts being produced around the third month of age in the pineal gland [5,7], or the initiation of cortisol secretion, which occurs from around eight weeks to nine months of age [8,9]. Melatonin has sleep-inducing activity, influences the regulation of the neuroendocrine system, and regulates the circadian rhythms and various physiological processes [10]. Melatonin synthesis depends on tryptophan availability and other nutritional factors, such as folate and vitamin B6 intake [11], as well as on light–dark cycle variation [12]. On the other hand, cortisol is a steroid or glucocorticoid hormone produced in the adrenal cortex, directed into the blood circulation after activation of the hypothalamic–pituitary–adrenal axis [13]. This hormone stimulates the physiological use of proteins and fats, regulates the glucose levels [14] and inflammation levels, contributes to memory and concentration, and controls the sleep–wake cycle [9,13].

The SCN synchronizes the biological rhythms influenced by “zeitgebers” (from the German “time givers”), which are external cues capable of modulating the circadian rhythm, such as food availability, temperature, exercise, and, most importantly, the light–dark cycle [1,2,15]. The synchronization of the central regulator and the external environment regulates the peripheral clocks by coordinating physiological processes (i.e., body temperature, hormone secretion, sleep–wake cycles) through autonomic nervous connections and the endocrine system. The peripheral clocks locally manage cellular processes (cell division, metabolism, local specific functions, etc.) and the harmonious relation between the central clock and the target tissue cellular clocks, providing an internal time marker [4,16].

At the molecular level, the SCN depends on a complex network of genes and proteins involving the internal pacemaker clock genes (CLOCK (or NPAS2), BMAL1, PER1, PER2, PER3, CRY1, CRY2), implicated in the autoregulation of the circadian rhythm. Surprisingly, the CLOCK and BMAL1/MOP3 genes activate the transcription of their own repressors (PER and CRY). Alterations in the clock genes lead to the dysregulation of metabolic processes [2,3].

**Figure 1:**
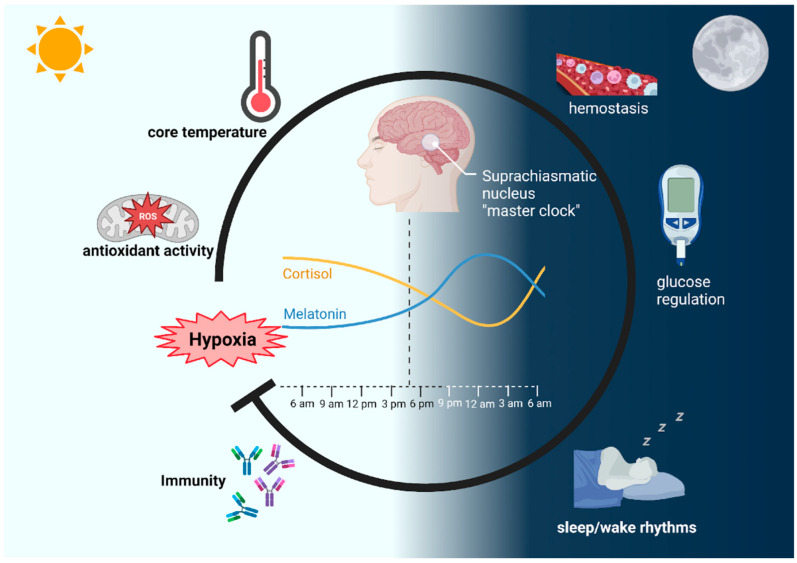
The circadian rhythm is a biological process that runs in a 24 h cycle, regulated by the suprachiasmatic nucleus in response to light–dark external cues. Many physiological systems are regulated by the circadian rhythm through the activity of cortisol and melatonin, which fluctuate throughout the day. Pathological states of chronic hypoxia can disrupt the circadian rhythm.

The circadian rhythm dictates physiological processes in multiple organ systems. in the respiratory system, studies showed that diurnal variations occur in airway resistance, lung capacity, gas exchange, and even respiratory symptoms. Disruptions in the circadian rhythm could alter these processes [17,18,19]. Interestingly, many chronic lung diseases (chronic obstructive pulmonary disease (COPD), asthma, and interstitial lung disease) are characterized by hypoxia, a state in which oxygen is not sufficient at the tissue level [20,21,22]. Multiple mechanisms can lead to hypoxia, including impaired lung function, a reduction in the oxygen diffusion capacity, or an imbalance in ventilation–perfusion, which can cause tissue damage [23], exacerbate respiratory symptoms (Figure 1) [24], and worsen patient prognosis [25]. Conversely, hypoxia also alters the circadian rhythm, suggesting that hypoxia plays an important role in the pathophysiology of chronic respiratory diseases. Therefore, a better understanding of the underlying mechanisms of such variations could improve the current treatment options.

## 2. The Impact of Hypoxia on Physiological and Cellular Circadian Rhythms

As mentioned before, hypoxia occurs when a tissue oxygen demand is greater than the oxygen received. Depending on how long this oxygen deficiency lasts, it can be acute or chronic. In an attempt at maintaining cellular homeostasis, hypoxia triggers several signaling mechanisms and physiological effects. Systemic hypoxia occurs in numerous diseased states, including lung disease, viral infections, inflammation, cancer, stroke, and ischemic heart disease. In chronic lung diseases, sustained hypoxia is caused by a reduced oxygen supply, as observed in obstructive sleep apnea (OSA) and chronic obstructive pulmonary disease (COPD), among others [20,21,22]. Interestingly, hypoxia alters the oscillations of body temperature, metabolic rate, and cortisol and melatonin release in humans. Additionally, in animals, hypoxia modifies the expression of the central clock gene in time- and tissue-dependent ways [26]. Intermittent hypoxia causes oxidative stress, mitochondrial dysfunction, inflammation, and overactivation of the sympathetic nervous system, among many other effects [24].

The daily rhythms are subject to regulation by multiple internal and external factors, including oscillations in oxygen levels. A hypoxic state can increase the activity of hypoxia-inducible transcription factor 1 alpha (HIF-1α), which modulates the circadian clock genes [27,28]. On the other hand, the activation of HIF-1α and HIF-2α regulates the physiological adaptation to sustained hypoxia, such as that experienced during prolonged stays at high altitudes [29]. A molecular regulatory relationship exists between oxygen levels and the circadian clock signaling pathway, given that HIF-1α, with BMAL1, directly binds to clock gene promoters, thus regulating the expression of clock genes. Inversely, clock proteins bind to the promoter of HIF1A, therefore regulating the expression of HIF-1α [30]. The balance between the HIF-1α/CLOCK pathways can be altered in states of hypoxia, including intermittent hypoxia, that modify the expression of HIF-1α, as occurs in OSA [30]. Interestingly, murine models showed that OSA disrupts the diurnal rhythms of cerebral arteries as well as the expression of clock genes [31].

Furthermore, hypoxia led to elevated levels of PER1 and CLOCK protein in mice [32], and in green monkey kidney fibroblasts (COS-1 cells) and mice, MOP3 (BMAL1) was shown to interact with HIF-1α and HIF- 2α [33]. Additionally, in cardiac muscle, HIF was shown to stabilize the circadian rhythm protein period 2 (PER2), an essential component in ischemic adaptation [34]. PER and HIF-1α have a structural overlap, containing the PER-Arnt-Sim (PAS) domain, suggesting a shared evolutionary origin and possible biological function. The PAS domain is involved in multiple processes that include signal transduction in response to external cue signaling (light, oxygen, etc.). Although the two proteins have independent functions, the presence of the PAS domain in their structure alludes to a crosstalk between them and to an interaction between the circadian rhythm and hypoxia [35,36]. In this context, in patients with type 2 diabetes, the level of metabolic control (measured through the levels of pyruvate and lactate in the presence of hypoxia induced by anaerobic glycolysis) was associated with the expression of clock genes, observing a positive correlation between HIF-1α and the clock genes [37]. Notably, it was observed in vivo in mice that hypoxia induced a shift in the phase of the circadian clock in a tissue-specific manner, causing intertissue asynchrony of the circadian clock [38]. Cryptochrome 1 (CRY1) is a negative regulator of HIF [39,40], interacting with the basic helix–loop–helix (bHLH) domain of HIF-1α and reducing the half-life of HIF-1α, which results in reduced binding of HIF to its gene promoters target and affects the response to hypoxia [39]. These studies demonstrated that hypoxia can modulate the circadian rhythm in vivo and in vitro [39,41]. Moreover, the acid produced during the cellular metabolic response to hypoxia suppresses the circadian clock through the decreased translation of clock constituents [42].

## 3. Pulmonary Pathologies and Their Relationship with the Circadian Cycle

The respiratory system is also subject to circadian rhythm regulation by the SCN, mainly mediated by the vagus nerve [43]. In addition to this central regulation, a “pulmonary clock” was also described, by examining the expression of clock genes in multiple tissues in mice and humans [43,44]. Disturbances in the circadian clock during chronic lung diseases could play a role in increased oxidative stress, the inflammatory response, metabolic imbalances, hypoxia/hyperoxia, mucus secretion, autophagy, and lung function disorders [17]. Proper synchronization and maintenance of the circadian rhythm are essential for normal lung function. An alteration in the circadian clock, due to genetic or environmental factors, produces stress [19]. For instance, exposure to cigarette smoke (CS) disrupts the normal circadian rhythm in the lungs [45,46].

An intrinsic physiological process such as aging is associated with alterations in the circadian rhythm. Aging results from accumulated damage at the molecular and cellular levels over time. The gradual decline in both physical and mental capacities leaves the organism vulnerable to diseases and death. In this sense, aging decreases the compliance of the chest wall and the respiratory system, as well as the elastic recoil of the lungs [47]. Moreover, hypoxia is a deleterious mechanism involved in the pathological processes of aging through the activity of HIF-1α as well as nuclear factor κβ (NF-κB) [48]. Considering that hypoxia is a key element of chronic respiratory diseases, in the following paragraphs, we aim to understand the interplay between the circadian rhythm, hypoxia, and aging in the pathophysiology of such diseases.

### 3.1. Asthma

Asthma is a heterogeneous disease characterized by chronic inflammation and airway hyperresponsiveness and remodeling resulting in a wide variety of clinical presentations [49]. Circadian rhythm disruption seems to influence asthma development and symptomology. The pulmonary function seems to be subject to day–night fluctuations, with an increase in airway resistance during sleep at night [50]. Some patients present nocturnal worsening of symptoms [51]. Multiple factors could contribute to this rhythmic worsening, including an increase in peak serum melatonin levels [52], the supine position, which can induce airflow obstruction [53], and air temperature cooling [54], among others. Night workers also seem to have an increased risk of developing asthma [55]. The inflammatory state also seems to be influenced by the circadian clock. Cortisol, an important immunosuppressor and anti-inflammatory hormone, shows daily fluctuations in its circulating levels, and its nadir (time point characterized by the lowest concentration) is reached at midnight. Moreover, children with asthma have lower circulating cortisol levels [56], and disturbances in the fluctuations of cortisol have been associated with nocturnal asthma severity [17,57].

**Figure 2:**
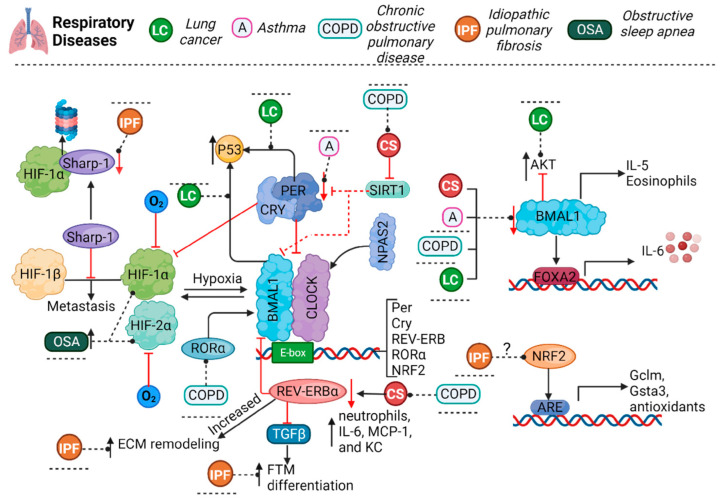
The molecular circadian clock involves an autoregulatory loop encompassing the transcription activators BMAL1, CLOCK and/or NPAS2, regulated by the repressors Per and CRY. During hypoxia, HIF-1α and HIF-2α activation increases. These molecules have a dual regulation: HIF-1α and BMAL1 bind together to clock gene promoters, while clock proteins bind to the promoter of HIF-1α. Per3 levels were found to be diminished in asthma. BMAL1 levels seem to be diminished in animal models or patients with asthma, COPD, or LC and are also diminished by CS exposure; inhibition of or decrease in BMAL1 can lead to an increase in IL-5 and IL-6 expression and in the number of eosinophils in the presence of asthma. COPD or CS exposure reduces SIRT1 expression, which regulates BMAL1 and PER2. Moreover, CS exposure in REV-ERBα KO mice increased the number of neutrophils and the expression of proinflammatory cytokines. In COPD, the levels of RORα, the counterpart to REV-ERBα and a regulator of BMAL1, are diminished. BMAL1 also regulates p53 expression, and PER binds to p53, regulating the stabilization and nuclear translocation of the protein. BMAL1 can also block the AKT signaling pathway, which is overactivated in lung cancer. Furthermore, the protein Sharp1 was shown to interact with HIF-1α to induce its proteasomal degradation and prevent its union with HIF-1β, which promotes the expression of metastatic genes. In IPF, the increment in REV-ERBα leads to ECM remodeling, and REV-ERBα suppression promotes fibroblast-to-myofibroblast differentiation. NRF2 is positively regulated by BMAL1-CLOCK, and NRF2 increases the expression of Gclm and Gsta3, proteins involved in glutathione metabolism. Additionally, Sharp1 expression is reduced in IPF. In OSA, HIFs are overactivated and increase the expression of Clock, BMAL1, and CRY2. A, asthma; COPD, chronic obstructive pulmonary disease; LC, lung cancer; IPF, idiopathic pulmonary disease; OSA, obstructive sleep apnea; O2, oxygen; BMAL1, brain and muscle ARNT-like 1; NPAS2, neuronal PAS domain protein 2; CLOCK, circadian locomotor output cycles kaput; PER, period; CRY, cryptochrome; SIRT1, sirtuin1; HIF, hypoxia-inducible factor; IL-5, interleukin 5; IL-6, interleukin 6; MCP-1, monocyte chemoattractant protein 1; KC, keratinocyte chemoattractant; AKT, protein kinase B; ECM, extracellular matrix; NRF2, nuclear factor erythroid 2-related factor 2; ARE, antioxidant response element.

Alterations in clock genes are associated with asthma severity. Asthmatic patients showed a significant downregulation of these genes [58,59]; specifically, PER3 levels appeared diminished in nocturnal asthma [59], and BMAL1 knock-out mice developed an asthmatic phenotype after viral exposure [58]. Interestingly, inhibition of the BMAL1/FOXA2 signaling pathway produced increases in nocturnal IL-6 levels in respiratory tract epithelial cells [60]. Furthermore, the induction of asthma was associated with a marked increase in lung inflammation in mice lacking BMAL1 expression in myeloid cells, increasing the number of eosinophils and IL-5 serum levels (Figure 2) [61].

### 3.2. Chronic Obstructive Pulmonary Disease

Chronic obstructive pulmonary disease (COPD), a progressive chronic respiratory disorder characterized by an obstructive ventilatory pattern [62], which unlike asthma is rarely reversible, is very often related to smoking or exposure to pollutants as well as to accelerated lung aging, leading to chronic respiratory failure [62,63]. Interestingly, COPD seems to follow a diurnal pattern, with patients reporting a substantial worsening of symptoms in the morning [62]. This could be related to the changes in peak expiratory flow (PEF), which is subject to circadian variations, peaking in the afternoon [64,65], while the maximum volume of air exhaled in the first second (FEV 1) is the lowest during the morning hours [66]. Moreover, in murine models, cigarette smoke (CS) was shown to reduce the activity of sirtuin 1 deacetylase (SIRT1), a marker of aging and inflammation, which regulates BMAL and PER2 through acetylation (Figure 2) [63,67,68].

The molecular clock seems to have a mutual relation with the inflammatory response and is altered in pathological states such as COPD. In human airway epithelial cells and lung tissue of REV-ERBα KO mice, CS exposure produced a heightened inflammatory response, with an increase in neutrophils and the release of proinflammatory cytokines (IL-6, MCP-1, and KC), in addition to the expression of p16 (a pro-senescence marker) and of markers of chronic lung remodeling, as observed in emphysema/COPD [69,70]. REV-ERBα is a nuclear heme receptor, a transcriptional repressor (binding and interacting with HDAC3 and NcoR), and a critical component of the molecular clock driving the daily rhythmicity of metabolism and inflammatory and immune responses [69]. In contrast to the antagonistic regulator REV-ERB, the retinoic acid-like orphan receptor alpha/gamma (RORα/γ) acts as an activator, and these molecules together are considered a “stabilizing loop” of the molecular clock [69]. Moreover, RORα levels are elevated in the lungs of COPD patients, but interestingly, RORα-deficient mice are protected against emphysema induced by elastase and CS exposure [71]. Interestingly, RORα responds to DNA damage, and a decrease in this protein is associated with low levels of apoptosis [67]. Interestingly, BMAL1 has also been shown to be underexpressed in human bronchial epithelial cells, lung tissue, peripheral blood mononuclear cells (PBMCs), sputum cell from COPD patients, and healthy cells exposed to CS [68,72,73]. The BMAL1 and CLOCK proteins seem to play an integral role in regulating cellular senescence, since the decrease in their expression by CS exposure upregulates senescence mediated by the MAPK pathways, a process implicated in the development of COPD (Figure 2) [73].

### 3.3. Lung Cancer

Lung cancer is the most commonly diagnosed cancer type in both sexes combined (11.6%) and the leading cause of cancer death among men [74]. Smoking has a causal link with the development of lung cancer, as shown by extensive epidemiologic research evidence [75,76,77]. In recent years, the relationship between sleep (duration, quality, and timing) and cancer risk has been consolidated particularly in regard to the development of lung cancer [78,79,80,81]. For instance, insomnia was causally associated with an increased risk of lung adenocarcinoma, while sleep with typical duration showed a protective effect on the risk of lung adenocarcinoma [82,83]. Also, clock genes, primarily BMAL1, have been implicated in tumorigenesis [84,85].

Clock genes’ behavior seems to depend on the type of lung cancer. In a recent study, the overexpression of CRY2, BMAL1, and RORα and the underexpression of Timeless and NPAS2 were associated with a favorable prognosis of lung adenocarcinoma. On the other hand, heightened expression of Sharp2 was observed in squamous cell carcinoma and was associated with poor survival. In the case of non-small cell lung cancer (NSCLC), clock genes establish a cancer circadian rhythm, asynchronic with respect to that of healthy tissues [86]. Moreover, CS exposure decreased Nr1d1 expression in both tumor-resistant and tumor-susceptible mice, making them more prone to developing CS-related lung cancer (Figure 2) [87].

The circadian rhythm also seems to regulate the expression of tumor suppressor genes and proto-oncogenes, such as p53, a critical tumor suppressor protein involved in the DNA damage response and apoptosis, whose transcription, stability, and activity are modulated by BMAL1 and PER2 [88,89,90]. While BMAL1 binds to the promoter region of p53, PER2 binds to the p53 protein, providing stabilization and nuclear translocation for its activation, in the presence of DNA damage in cells [88,89,90,91,92,93]. Furthermore, BMAL1 suppresses cell invasion by blocking the PI3K-AKT-MMP-2 pathway, as seen in BMAL1 KO human lung cancer and glioma cell lines [94]. The PI3K-AKT-MMP-2 pathway is involved in a myriad of cellular processes that can be altered in cancerous environments, including cellular apoptosis, migration/invasion, cell growth, and angiogenesis, among others, through the regulation of FOXO, mTORC1/2, XIAP, Bcl2, and MDM2, the last of which inhibits p53 activity (Figure 2) [95].

Melatonin is also an important regulator of oncogenic processes, especially those induced by hypoxia, and could serve as a potential therapeutic agent. Among the key anti-cancer mechanisms of melatonin are the inhibition of hypoxia-induced proliferation in cancer cells, the induction of apoptosis, anti-angiogenesis mechanisms, the inhibition of HIFs, the reduction in hypoxia-induced reactive oxygen species (ROS), the inhibition of cancer cell migration and invasion, the attenuation of epithelial–mesenchymal transition (EMT), the inhibition of endoplasmic reticulum stress, the reversion of altered states of metabolism (e.g., the Warburg effect), and the improvement of chemoradiotherapy sensitivity [96,97,98].

NSCLC is associated with an overactivation of AKT [99]. In a study with 1144 patients with such a disease, 3.7% of the patients were identified to have PIK3CA mutations in exons 9 and 20, and up to 57.1% of the patients showed additional aberrations in oncogenic drivers [100]. Furthermore, the gene ARNTL (that encodes the BMAL protein) was found to protect against lung adenocarcinoma growth, and the ARNTL gene was shown to increase the expression of the circular RNA circGUCY1A2 and activate the miR-200c-3p/PTEN axis, initiating a tumor suppressive activity [101].

The proteins differentiated embryonic chondrocyte expressed gene 1 (DEC1; BHLHE40/Stra13/Sharp2) and 2 (DEC2; BHLHE41/Sharp1), related to the circadian genes, can act as repressors and coactivators of BMAL1 and CLOCK or NPAS [102]. These two proteins are implicated in regulating cell differentiation, circadian rhythms, apoptosis, the response to hypoxia, EMT, and carcinogenesis [103,104]. As stated, HIFs are activated during hypoxia and are described to participate in different aspects of cancer pathophysiology. Interestingly, Sharp1 interacts with HIF-1α, driving it to proteosome degradation and preventing the formation of the HIF-1α/1β heterodimer that promotes metastatic gene expression [105]. Sharp1 suppresses metastasis in various types of tumors including breast, endometrial, prostate, thyroid, and lung cancers [106,107,108,109,110]. Paradoxically, in human breast cancer cells, HIF-2α was shown to increase Sharp1 expression through the activation of AKT in a hypoxic state, which in turn promoted c-Myc expression and favored cellular proliferation (Figure 2) [104].

Concerning the antitumor effects, the overexpression of Sharp1 inhibited the formation of colonies of lung cancer cells (A549, NCI-H520, and NCI-H596 cells) by promoting a decrease in cyclin D1 (CCND1) expression [109]. In this sense, Sharp1 and Sharp2 seem to have opposing roles in cancer progression. While Sharp1 was shown to suppress EMT and metastasis by attenuating NOTCH1 signaling in endometrial cancer cells [111] and TGF-β-associated EMT in prostate cancer PC-3 cells [110], Sharp2 favored TGF-β-associated EMT [103] and interplayed with NOTCH1 to favor cell growth and invasiveness [110]. Furthermore, in fibroblasts, Sharp1 was activated during DNA damage and was involved in cell cycle arrest and apoptosis; an aberrant expression of Sharp1 could thus participate in tumorigenesis [112]. Unexpectedly, in various cancer cell lines, the hypoxia-induced expression of both Sharp1 and Sharp2 downregulated the MLH1 protein, an essential component of the DNA mismatch repair (MMR) system [113]. However, as stated, Sharp1/2 can have both repressor and oncogenic activity, and their function seems to vary by tissue type [114].

### 3.4. Idiopathic Pulmonary Fibrosis

Idiopathic pulmonary fibrosis (IPF) is a chronic, progressive lung disease primarily affecting the elderly, characterized by irreversible damage and an unknown etiology, which offers limited treatment options. Typically, patients diagnosed with IPF face a grim prognosis, succumbing to respiratory failure within two to five years [115,116,117]. The pathogenesis of IPF involves the activation of epithelial cells in response to various injuries, leading to the release of mediators that contribute to fibrosis. These mediators include cytokines and growth factors, notably, platelet-derived growth factor (PDGF), transforming growth factor β (TGF-β), tumor necrosis factor α (TNFα), endothelin-1 (ET-1), and connective tissue growth factor (CTGF) [118]. Particularly, TGF-β1 plays a central role in fibrotic tissue formation within the lungs, which is a key driver of IPF development [119]. TGF-β1 promotes fibroblast-to-myofibroblast differentiation, EMT, and the production of matrix metalloproteinases, all contributing to the formation of fibroblast foci [120]. These foci generate excessive extracellular matrix deposition, predominantly, collagen deposition, leading to lung architectural scarring and destruction [118,121,122,123,124]. Circadian oscillations within the lung epithelium were associated with inflammatory responses in IPF patients [125]. These oscillations are implicated in the remodeling of the lung’s extracellular matrix, largely due to increased levels of the REV-ERBα protein, a negative regulator of BMAL1, which itself contributes to the regulation of inflammatory mediators [126].

In a mouse model of bleomycin-induced pulmonary fibrosis, it was observed that the REV-ERBα protein, involved in circadian cycle regulation, inhibited the fibrotic response triggered by TGFβ. Conversely, the suppression of REV-ERBα promoted fibroblast-to-myofibroblast (FTM) differentiation, driving fibrosis [126], while REV-ERBα agonists prevented TGFβ-induced FTM, reduced the IL-6 levels, and reduced the expression of extracellular matrix (ECM) genes [127]. In mice, bleomycin exposure lowered the REV-ERBα levels, which was reverted with a REV-ERBα agonist (SR9009), and prevented collagen overexpression, while influenza A virus (IAV) infection increased the collagen and lysyl oxidase levels, which was prevented by a REV-ERBα agonist (GSK4112) [128]. Additionally, the circadian transcription factor BMAL1 is known to regulate genes through E-box elements in their promoters [129]. There is evidence to suggest that BMAL1 controls ECM genes and participates in FTM differentiation mediated by TGF-β1 [130,131]. The precise relationship between fibrosis and the circadian cycle remains unclear and raises several questions (Figure 2).

Nevertheless, the inactivation of BMAL1 in animal models resulted in a complete loss of circadian rhythm under constant darkness conditions [132]. In other studies, circadian clocks were identified as endogenous regulators of protective responses to oxidative stress by controlling the transcription factor NRF2 in the mouse lung. The transcription of NRF2 is positively regulated by the circadian transcription factors CLOCK and BMAL1 through a temporal control of E-box elements in the NRF2 gene promoter. This results in increased NRF2 protein levels and circadian transcription of key antioxidant genes, such as Gclm and Gsta3, which are involved in glutathione metabolism. NRF2 periodic activity is critical for coordinating protection against oxidative tissue damage and fibrotic injury over time. The disruption of circadian rhythms in a bleomycin mouse model was associated with increased oxidative protein damage and a spontaneous fibrotic phenotype in the lungs (Figure 2) [133].

A reduced expression in the BHLHE41 gene [134] was observed in hypoxic lung fibroblasts obtained from IPF individuals. As mentioned earlier, the protein encoded by this gene is recognized as a tumor suppressor. Consequently, the absence of its expression in the lung fibroblasts of IPF patients led us to hypothesize that BHLHE41 may play a significant role in fibrosis progression. Such a hypothesis gains credence considering the observation that TGF-β induces the upregulation of BHLHE40 and the downregulation of BHLHE41 in prostate cancer cells (PC-3) [103]. It is worth noting that TGF-β triggers the phosphorylation of Smad2, subsequently leading to the activation of mesenchymal markers such as N-cadherin and vimentin, while concurrently suppressing epithelial markers like E-cadherin [103]. This suggests that TGF-β may initiate EMT, a process known to be involved in developing pulmonary fibrosis. Furthermore, evidence indicates that the two genes BHLHE40 and BHLHE41 may have opposing effects in the context of cancer development.

### 3.5. Obstructive Sleep Apnea

Obstructive sleep apnea (OSA) is the most common type of sleep-related breathing disorder, characterized by repeated episodes of complete or partial airway collapse in association with a decrease in oxygen saturation or with arousal from sleep. These episodes lead to fragmented and nonrestorative sleep that, over time, impact negatively on cardiovascular health, cognitive performance, and quality of life [135,136]. Apnea is defined as an interruption of airflow for at least ten seconds, resulting in a complete lack of breathing; these events are obstructive, central, or mixed. Obstructive apneas occur when the airway is blocked, but respiratory effort is present in the thorax and abdomen. In central apneas, there is no respiratory effort or airflow, indicating that the brain is not sending signals to stimulate breathing. Mixed apneas are a combination of these two dysfunctions [136,137,138,139]. The standard diagnosis for OSA involves nocturnal polysomnography, which records the number of apneas and hypopneas per hour of effective sleep. The Apnea–Hypopnea Index (AHI) is used to assess the severity of the condition [140,141,142,143]. The main symptoms of OSA include loud snoring, nighttime awakenings, daytime sleepiness, and fatigue [135,143]. Possible additional signs include morning headaches, memory and concentration problems, night sweats, and wheezing [136]. OSA patients often experience cycles of oxygen desaturation followed by reoxygenation during sleep [144,145]. States of hypoxia, such as the prolonged period of hypobaric hypoxia present during long-duration flights, are shown to disrupt multiple processes regulated by the circadian rhythm, including the maintenance of core body temperature [146], melatonin secretion [147], and the cortisol secretion cycle [148]. These changes significantly modify sleep (latency, time, and quality of sleep) and can be influenced by individual characteristics such as age, physical fitness, and the sympathetic reaction to hypoxia.

To provide further support for this association, bidirectional interactions between HIF-1α and the circadian clock have been demonstrated. For instance, BMAL1 and CLOCK form heterodimers and regulate the rhythmic expression of HIF-1α [41]. Moreover, in a hypoxic environment, alterations in the expression of circadian genes were observed. Overexpression of HIF-1α and HIF-2α was shown to increase the gene expression of CLOCK, BMAL1, and CRY2, while decreasing the expression of PER1, PER2, PER3, CRY1, and CKIε [149]. In a separate study involving individuals with obstructive sleep apnea (OSA), it was noted that the transcription of BMAL1, CLOCK, and CRY2 was irregular, and the levels of CRY1 and PER3 significantly decreased at midnight. This suggests that the latter two genes could potentially serve as prognostic markers for OSA severity (Figure 2) [150]. Although the role of circadian gene polymorphisms in respiratory pathologies is relatively unexplored, recent findings suggest their potential significance. For instance, a study revealed that mutations in the BHLHE41 gene were associated with reduced total sleep [151]. However, the implications of such mutations in respiratory diseases remain unclear. Therefore, investigating whether these mutations, as well as those in other circadian genes, could be linked to various types of OSA is an intriguing avenue for future research. Moreover, sleep and circadian rhythms become impaired in aging; particularly, OSA is more frequent in older adults due to age-related alterations in the upper airway, causing sleep fragmentation and hypoxemia. This evidence suggests that sleep disorders, aging, and hypoxia are closely related and that it is necessary to perform further studies to unveil the causes and the directionality of the events that result in alterations in the circadian rhythms.

### 3.6. Influenza and COVID-19

Influenza, a highly contagious and often fatal disease, claims nearly half a million lives annually worldwide. Until now, four distinct types of influenza viruses have been recognized: A, B, C, and D. Only types A and C viruses are recognized to infect humans. Types A and B viruses co-circulate as the primary seasonal strains, giving rise to a spectrum of respiratory infections and related complications, ranging from mild to severe, in human populations. The typical influenza symptoms encompass fever, fatigue, cough, and body aches [152,153,154,155]. Almost 10% of the global population falls prey to influenza each year [152,154], since this virus incubates for a period ranging from 1 to 4 days [156].

Lung damage inflicted by influenza is associated with disruptions in the circadian clock, compromised lung function, and decreased survival rates [157]. The protein BMAL1 plays a pivotal role in alveolar epithelial cells (AECs), with specific BMAL1 deletion disrupting lung neutrophil infiltration, biomechanical functions, and the response to influenza infection [158]. Conversely, the genetic deletion of BMAL1 and the overexpression or activation of REV-ERB using synthetic agonists inhibit the replication of hepatitis C virus (HCV) and related flaviviruses like dengue and Zika. The overexpression of REV-ERB denies HCV cell entry, viral RNA replication, and the release of infectious particles through lipid signaling pathway disruption [159]. REV-ERB agonists inhibit HIV transcription and replication in vivo in primary cell lines [160]. Together, these findings indicate the important role of circadian genes in viral infections and their influence on immune responses.

In recent times, the world endured one of its most devastating pandemics, resulting in the loss of millions of lives due to a Severe Acute Respiratory Syndrome Coronavirus 2 (SARS-CoV-2) outbreak, responsible for Coronavirus Disease 2019 (COVID-19) [161,162]. During the COVID-19 pandemic, two factors significantly impacted sleep quality: irregular schedules and confinement [163,164]. Such disruptions altered the daily routines, including office and work schedules, social engagements, and recreational activities [163]. It is well documented that even a single night of sleep deprivation can lead to mood swings and a weakened immune system [163,165]. This connection could result in disturbances in mitochondrial metabolism and influence the normal functions of the immune response [166]. Interestingly, several viruses inhibit mitochondrial melatonin production [166]; notably, the loss of circadian activation coincides with suppressed REV-ERBα expression, indicating a possible link between clock genes and inflammatory pathways [167].

The circadian clock influences both innate and adaptive immune systems, impacting processes from leukocyte mobilization and chemotaxis to cytokine release and T-cell differentiation [168]. The pathogenicity of viral infections can be influenced by the host’s circadian clock through two primary mechanisms: (1) a direct regulation of viral replication within the target cells; (2) indirect effects on innate and adaptive immune responses [169,170,171]. Intriguingly, recent studies concluded that the circadian clock, via REV-ERBα and BMAL1, significantly influences the airway inflammatory responses [125,172]. Circadian cycle genes regulate cytokines, including IL-6, a function that exhibits diurnal variation and has implications in inflammatory processes, as seen in patients with rheumatoid arthritis [173]. Such a relationship could also shed light on pharmacological treatments variability; for instance, the effectiveness of glucocorticoids depends on the intact functioning of the circadian clock in the airways [125]. Therefore, viral infections, inflammatory responses, and circadian clock genes appear to share intricate pathways, and comprehending these connections may enhance our understanding of the pathogenesis of such infectious diseases.

## 4. Understanding Hypoxia and Circadian Rhythm Genes from a Systems Biology Perspective

As seen in the previous sections, there is an unequivocal interplay between the circadian rhythm and hypoxia. In fact, several genes are involved in respiratory diseases (BMALl1, FOXa2, CKIε, PER3, TIM, RORα, Clock, PER2-3, BHLHE40-41, REV-ERBα, and CRY1-2). Thus, we built a structural network with the Cytoscape software version 3.8.0.; as input data we used the set of genes mentioned above and the results obtained from the gene association analysis performed on the Comparative Toxicogenomics Database (CTD), a public database that provides curated information about chemical–gene/protein interactions and chemical–disease and gene–disease relationships (https://ctdbase.org/, accessed on 14 September 2023).

**Figure 3:**
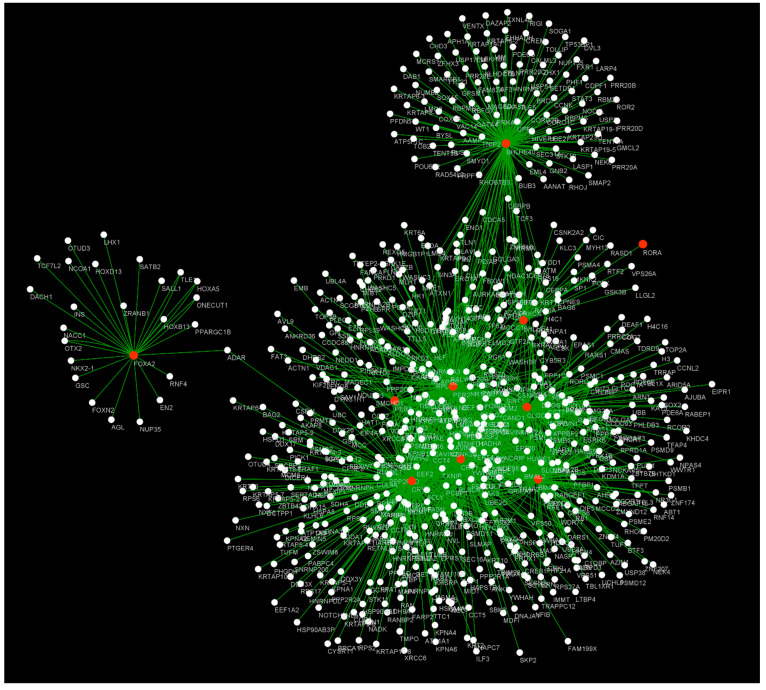
Network biology of hypoxia and circadian rhythm genes. This network represents the interaction between the hypoxia and circadian rhythm genes (orange nodes) and the genes reported to be associated with hypoxia and circadian rhythm genes (white nodes). Each edge (green) represents an independent reported association by CTD (https://ctdbase.org/, accessed on 14 September 2023). BMAL1, FOXa2, CKIε, PER3, TIM, Clock, PER2-3, BHLHE40-41, REV-ERBα, and CRY1-2 are hubs in this network, while RORα stands alone.

However, the best way to improve the understanding of such interplay is by systems biology. As seen in Figure 3, in the center of the network appear the BMAL1, CKIε, PER3, TIM, Clock, PER2-3, BHLHE41, REV-ERBα, and CRY1-2 genes, while BHL-HE40-41 and FOXA2 stand apart from the rest of the genes, both genes being hubs (nodes of the network with high centrality [174]) in the network.

Additionally, we complemented the structural network analysis with a gene enrichment analysis using GeneMANIA (https://genemania.org/, accessed on 14 September 2023), a Cytoscape plugin that identifies the most related genes using a large database (DAVID/GO) of functional interaction networks from multiple organisms and in which each related gene is traceable to the source network used to make the prediction [175]. The data suggested a wide interaction and a potential modulation of other genes that are also implicated in positive regulation of transcription, proteasome, regulation of telomerase RNA localization to Cajal bodies, regulation of splicing, regulation of protein localization to telomeres, viral carcinogenesis, cell cycle, ubiquitin-mediated proteolysis, and Wnt signaling pathways (Table 1).

When analyzing the genes from the structural network (above), we found that most of them are significantly associated with the cell cycle, circadian rhythms, transcription regulation, and proteolysis. Simultaneously, we found that viral carcinogenesis genes, splicing genes, and telomerase localization genes are quite associated with some of them. Moreover, we found that Wnt signaling pathways are enriched on this gene set; such pathways are involved in cell surface receptor regulation and are clinically associated with the development of some types of cancer, such as melanoma and prostate and lung cancer, and aging [176,177,178,179]. Additionally, Wnt signaling is upregulated in individuals with idiopathic pulmonary fibrosis (IPF) [180,181], suggesting a strong connection between age-related diseases and such signaling pathways. Therefore, we found a tight relation between certain pathologies and such genes, which may shed light on the causal mechanisms linking some of these diseases with the circadian clock.

## 5. Interactions between Aging, Hypoxia, and Circadian Rhythms

The prevalence of lung diseases such as idiopathic pulmonary fibrosis (IPF), chronic obstructive pulmonary disease (COPD), and acute lung injury was found to increase considerably with age [182]. Interestingly, the prevalence of IPF and COPD increases almost three times in individuals older than 60 years [183]. Similarly, sleep-related breathing disorders such as OSA increase with age, and the combination of this condition with chronic lung diseases causes a progressive impairment of the normal lung function, marked structural changes, impaired gas exchange, hypoxia, immunologic changes, and vulnerability to infections (immunosenescence) [184]. Interestingly, a recent single-cell transcriptional analysis suggested that normal chronologic aging increases signaling pathways mainly associated with cholesterol biosynthesis in type-2 pneumocytes A and lipofibroblasts, as well as the number of epithelial cells, a hallmark of lung aging [185].

On the other hand, considering that hypoxia is a key element in altering the circadian rhythm genes, it is also important to include the interplay of this effect with hypoxia induced by chronic respiratory diseases and sleep-related breathing disorders. In this sense, one of the key molecular regulators of hypoxia is the hypoxia-inducible factor (HIF) pathway. HIF-1α is a transcription factor that activates genes involved in a variety of cellular responses to hypoxia, including angiogenesis (the formation of new blood vessels), metabolism, and cell survival. In this context, the activity of HIF-1α as well as NF-κB during aging participates in the progression of age-related diseases [48]. Interestingly, the overexpression of the circadian clock BMAL1 gene can inhibit the expression of the HIF-1α protein [186].

Among the changes related to aging, HIF-1α induces a deficit in mitochondrial biogenesis, which impairs energy-dependent cellular processes, including tissue repair pathways [187]. Mitochondrial dysfunction can lead to the accumulation of ROS, which can damage the DNA and disrupt the circadian clock function. In this context, mitochondrial proteins can adversely modify acetyl groups produced by fat and glucose metabolism alterations associated with age via the mitochondrial deacetylase Sirt3. Nevertheless, perturbation of such protein leads to inflammasome activation and to the chronic inflammation associated with aging known as *inflammaging* [188]. Inflammation, in turn, can activate the NF-κB transcription factor, which can repress the transcription of the clock genes; this process becomes a cycle, since the circadian clock regulates the expression of genes involved in DNA repair and in the oxidative stress response.

In summary, a number of molecular connections between aging, hypoxia, and circadian clocks were identified. For example:

(a) HIF can regulate the expression of clock genes, and vice versa.
(b) The circadian clock regulates the expression of genes involved in hypoxia signaling.
(c) Mitochondrial dysfunction, which is a common feature of aging, can disrupt both circadian clock function and hypoxia signaling.
(d) Inflammation, which is another common feature of aging, can also disrupt both circadian clock function and hypoxia signaling.

These findings suggest a complex network of interactions between aging, hypoxia, and circadian rhythms. By understanding these interactions, we may be able to develop new strategies to delay or prevent the onset of age-related diseases.

## 6. Conclusions

Altogether, the evidence summarized in the present review suggests a complex network of interactions between aging, hypoxia, and circadian rhythms. By understanding these interactions, we may be able to develop new strategies to delay or prevent the onset of age-related diseases. In the natural aging process, lung function undergoes a gradual decline, while airflow limitation induces hypoxia associated with age [73]. Additionally, age impacts the circadian rhythms, and any disruptions in these cycles can trigger alterations in lung function, potentially fostering cellular senescence-associated oxidative stress due to mitochondrial dysfunctions and epigenetic modifications, including those involving miRNAs [73,189]. Such alterations may have significant implications for the development of various diseases [190,191], including several types of cancer, viral infections, and many others briefly discussed in the present review. Overall, the molecular connection between aging, hypoxia, and circadian rhythms is complex and multifaceted. However, research in this area is rapidly advancing, and we are gaining a better understanding of how these factors interact to influence the aging process.

## Figures and Tables

**Table 1:** Enrichment analysis of hypoxia- and circadian rhythm-associated genes.

Pathway	*p*-Value	Database
Regulation of circadian rhythm	1.1 × 10^−9^	DAVID/GO—CTD
Positive regulation of transcription	6 × 10^−21^	DAVID/GO
Proteasome	3.8 × 10^−8^	DAVID/KEEG—CTD
Regulation of telomerase RNA localization to Cajal bodies	1.7 × 10^−7^	DAVID/GO
Regulation of splicing	9.1 × 10^−5^	DAVID/GO
Regulation of protein localization to telomere	1.7 × 10^−7^	DAVID/GO
Viral carcinogenesis	6.0 × 10^−10^	DAVID/KEEG
Cell cycle	4.5 × 10^−25^	DAVID/KEEG—CTD
Ubiquitin-mediated proteolysis	2 × 10^−5^	CTD
Wnt signaling pathways	6 × 10^−5^	CTD

## Data Availability

Not applicable.
